# Dendritic cell combination therapy reduces the toxicity of triptolide and ameliorates colitis in murine models

**DOI:** 10.1080/10717544.2022.2044935

**Published:** 2022-02-28

**Authors:** Quan Rao, Guang-chao Ma, Hao Wu, Meng Li, Wei Xu, Guo-jun Wang, Dong Wang, Cong-en Zhang, Zhi-jie Ma, Zhong-tao Zhang

**Affiliations:** a Department of General Surgery, Beijing Friendship Hospital, Capital Medical University, Beijing, China; b Beijing Key Laboratory of Cancer Invasion and Metastasis Research & National Clinical Research Center for Digestive Diseases, Beijing, China; c Department of Pharmacy, Beijing Friendship Hospital, Capital Medical University, Beijing, China; d Chinese Materia Medica, Yunnan University of Chinese Medicine, Kunming, China

**Keywords:** Triptolide, toxicity, dendritic cell, ulcerative colitis, tolerogenic

## Abstract

Triptolide (TP) exerts a promising effect in the treatment of ulcerative colitis (UC). However, its toxicity seriously hinders its application in the clinic. Previous studies indicated that dendritic cells (DCs) are the main target through which TP exerts its immunoregulatory effect. Thus, we designed an approach to target DCs *in vitro* to avoid the direct exposure of organs to TP. Our results revealed that DCs pretreated with TP (DCTP) exerted satisfactory therapeutic effects in mice with colitis, resulting in improved colonic inflammation and alleviated local lesion damage. In addition, no obvious toxicity was observed. DCTP also reshaped the immune milieu by decreasing CD4^+^ T cell numbers and increasing regulatory T cell numbers in the spleen, mesenteric lymph nodes, peripheral blood and colon; these effects were further confirmed *in vitro*. Downregulation of CD80/86, ICAM-1, MHCI, TLR2/4, TNF-α, and IL-6 expression and upregulation of programmed cell death ligand 1 (PDL1) and IL-10 expression were observed, indicating that DCs were converted into tolerogenic DCs. In conclusion, DCTP can effectively reduce toxicity and alleviate colonic inflammation and local lesion damage in mice with colitis. The immune mechanism underlying the effects of DCTP included the conversion of DCs into tolerogenic DCs and the alteration of T cell differentiation to produce immunoinhibitory rather than immunostimulatory T cells.

## Introduction

Ulcerative colitis (UC) is an idiopathic inflammatory disease, and its global incidence has been increasing in recent years. UC mainly afflicts young adults and patients suffer from relapsing and remitting clinical presentations including hematochezia, diarrhea, mucous discharge, abdominal pain, tenesmus, fatigue, fevers, and weight loss (Hoivik et al., 2013). The repeated attacks and the lifelong course of this disease seriously diminish quality of life and cause great psychological and physical pain in patients. Some risk factors, such as family history, gut microbiota, history of gastroenteritis, drugs (oral contraceptives, hormone replacement therapy, and nonsteroidal anti-inflammatory drugs), and residence in urban areas, have been shown to be related to UC. Studies have revealed possible pathological factors that contribute to UC, including mucosal and epithelial barrier defects, dysbiosis, immune cells, and immune factors (Ungaro et al., 2017). Currently, drugs for UC treatment mainly include aminosalicylates, steroids, immunosuppressants, microecologics, and biological drugs, and these treatments are mainly focused on relieving symptoms. The lack of specific drugs for downstaging and the adverse effects caused by current drugs result in limited therapeutic benefit for patients (Kobayashi et al., 2020). Therefore, other approaches are urgently needed.

Triptolide (C_20_H_24_O_6_, TP), one of the most important extracts from *Tripterygium wilfordii* Hook F, shows effective anti-inflammatory and immunosuppressive properties in the contexts of autoimmune disease and transplant rejection (Matta et al., 2009; Han et al., 2012). Our previous study also showed that TP could accelerate the recovery of the microbiota and exert good therapeutic effects in mice with UC (Wu et al., 2019). Further studies have shown that TP mainly targets dendritic cells (DCs) to induce immunosuppression *in vivo* (Zhu et al., 2005; Zhang & Ma, 2010). Liu et al. (2007) found that TP inhibited lipopolysaccharide (LPS)-triggered upregulation of chemokine (C-C motif) receptor 7 (CCR7) expression and prostaglandin E_2_ (PGE_2_) production by inhibiting cyclooxygenase-2 expression in DCs, which impaired DC migration and induced T cell tolerance.

However, studies have also shown that TP causes serious damage to multiple systems and organs including the liver, kidneys, heart, and reproductive system. The narrow therapeutic window of TP greatly limits its clinical application (Li et al., 2014).

Here, we examined the feasibility and functionality of TP combined with DCs in the treatment of experimental colitis. DCs were exposed to TP *in vitro* and subsequently injected into mice. The results showed that DCs pretreated with TP (DCTP) elicited immunosuppression *in vivo* and reduced TP toxicity. Notably, a therapeutic effect was achieved by DCTP treatment in mice with colitis, and the colonic immune microenvironment was reshaped, as shown by reduced T cell numbers and increased regulatory T cell (Treg) infiltration. Our study also revealed that DCs were converted into tolerogenic DCs by TP, and these tolerogenic DCs exhibited low expression of costimulatory factors and Toll-like receptors (TLRs), high expression of programmed cell death ligand 1 (PDL1) and altered expression of secretory cytokines. In summary, our findings provide evidence that TP combined with DCs is a promising treatment strategy for UC. Moreover, this study provides a new approach for the treatment of other autoimmune diseases and for the reduction of TP toxicity.

## Materials and methods

### Mice and cell line

Wild-type C57BL/6 (H-2^b^) mice (6–8 weeks old, male) were used in all the experiments (10 mice were used in each group for the UC model studies, and the experiments were repeated at least three times unless otherwise specified). All the animal experiments were carried out in the Laboratory Animal Center of Capital Medical University. All the animals were raised in accordance with the National Institutes of Health Guide for Laboratory Animals. The study was approved by the Animal Ethics Committee of Capital Medical University (certification number SCXK-JING 2016-0002). The murine cell line DC2.4 (H-2^b^) was cultured as described in our previous study (Rao et al., 2016). Briefly, DC2.4 cells were cultured in Dulbecco’s modified Eagle’s medium supplemented with 1% antibiotics, 1% glutamine, 1‰ β-mercaptoethanol, and 10% fetal bovine serum (FBS, Sigma, St. Louis, MO).

### Establishment of an experimental colitis model

Fifty male mice were randomly divided into five groups (*n* = 10/group, in the control group, model group, TP group, DC group, and DCTP group). After 1 week of adaptive feeding, the mice were fasted for 24 hours, and then, the mice were administered a single enema of 2,4,6-trinitrobenzene sulfonic acid (TNBS) solution (100 µL, 5% TNBS:ethanol = 1:1) or saline (control group); these solutions were administered with a catheter inserted 3–4 cm through the anus under light ether anesthesia as described in the study by D'Argenio et al. (2006). Then, the mice were inverted for 30 s to prevent solution outflow. On day 3, DCs preconditioned with an optimal dose of TP (15 ng/mL, 24 hours) were injected intravenously (DCTP group, 4 × 10^6^ DCTP per mouse) into mice with colitis. The mice in the control group (Con) and TNBS group (Mod) were administered normal saline. The mice in the other two groups received TP (China Food and Drug Control Institute, 111567, Beijing, China, TP group, 0.25 mg/kg) or 4 × 10^6^ DCs (DC group). Repeated injection with the same solution or cells was performed on days 6 and 9.

The weights of the mice were recorded daily. The disease activity index (DAI) was calculated as described in a previous study (Murano et al., 2000). Briefly, DAI=(weight loss score + fecal trait score + blood test score)/3.

The mice were sacrificed one day after the last injection. Peripheral blood samples were collected retro-orbitally into trisodium citrate tubes and normal tubes to obtain peripheral blood monocytes and serum. Complete colon tissues from the anus to the end of the cecum were harvested and measured. Partial spleen and mesenteric lymph nodes (mLNs) samples were harvested, and cells were isolated for examination by flow cytometry (FCM). Other organs, including the liver, kidneys, heart, testis, spleen, and colon, were also harvested and fixed with 4% paraformaldehyde.

### Histological examination

Colon and other tissues were stained with hematoxylin and eosin (H&E) for histological examination. The colonic histopathology score was evaluated by two double-blinded pathologists and the criteria were as follows: 0 points, normal and no inflammatory cell infiltration; one point, mild inflammatory cell infiltration but no damage to submucosal tissues; two points, moderate inflammatory cell infiltration and submucosal tissue destruction (injury range 10–25%); three points, obvious inflammatory cell infiltration, submucosal tissue destruction, and thickening of the colon wall (injury range 25–50%); and four points, severe inflammatory cell infiltration, severe colon tissue damage (>50% lesion range), and thickening of the colon wall (Boirivant et al., 2001).

### Evaluation of TP toxicity

The toxicity to the liver, kidneys, heart, and testis was examined by H&E staining. Furthermore, alanine aminotransferase (ALT) and aspartate aminotransferase (AST) levels were measured with the ALT and AST Assay Kit (Nanjing Jiancheng Bioengineering, Nanjing, China). The creatinine (Cr), lactate dehydrogenase (LDH), and creatine kinase MB (CK-MB) levels were measured with a Hitachi automatic biochemical analyzer 7600 (Shiga, Japan).

### Immune milieu in the peripheral blood, spleen, mLN, and colon

Peripheral blood was collected into trisodium citrate tubes, followed by treatment with Lymphoprep solution (TBD, Tianjin, China) to generate peripheral blood monocyte suspensions. Splenocytes and lymph node cells were isolated by grinding, filtering, and removing red blood cells with ACK lysis buffer. The mixture of lymphocytes was stained with rat anti-mouse monoclonal antibodies including fluorescein isothiocyanate-labeled anti-CD3e (Thermo, Waltham, MA), allophycocyanin-labeled anti-CD8a (Biolegend, San Diego, CA), phycoerythrin-labeled anti-CD4 (Thermo, Waltham, MA), and anti-FOXP3 (True-Nuclear™ Transcription Factor Buffer Set, Biolegend, San Diego, CA) antibodies, followed by FCM analysis. The levels of tumor necrosis factor (TNF)-a (Thermo, Waltham, MA), interleukin (IL)-1β (Thermo, Waltham, MA), IL-2 (Thermo, Waltham, MA), IL-6 (Thermo, Waltham, MA), and interferon (IFN)-γ (Thermo, Waltham, MA) in the serum were measured with enzyme-linked immunosorbent assays (ELISAs). T lymphocytes in the spleen and colon were also stained to measure the CD3 (Servicebio, Wuhan, China), CD4 (Servicebio, Wuhan, China), and forkhead box P3 (FoxP3) (Servicebio, Wuhan, China) levels by immunohistochemistry (IHC).

### Preparation of DCTP and measurement of DC surface molecule and secreted cytokine levels

DC2.4 cells were pretreated with TP at different concentrations ranging from 5 ng/mL to 40 ng/mL for 24 hours and then, cell viability was measured with a Cell Counting Kit-8 (CCK-8, Abcam, Cambridge, UK) to determine the optimal TP concentration. The optimal concentration was one that maintained a balance of DC proliferation and death, with the DC numbers remaining the same as the initial fixed cell number (con). Then, DC2.4 cells were pretreated with the optimal concentration of TP (15 ng/mL) to produce DCTP. DCs were stained with rat anti-mouse monoclonal antibodies, including fluorescein isothiocyanate-labeled anti-CD80 (eBioscience, San Diego, CA), anti-CD86 (Thermo, Waltham, MA), anti-intercellular adhesion molecule 1 (ICAM-1, Thermo, Waltham, MA), anti-MHC class I (MHCI, Thermo, Waltham, MA), and phycoerythrin-labeled anti-PDL1 (Thermo, Waltham, MA). The expression levels of surface molecules, including PDL1 (Abcam, Cambridge, UK), TLR2 (Abcam, Cambridge, UK), and TLR4 (Bioss, Beijing, China), were also measured by western blotting. The concentrations of secreted cytokines including IL-6 (Thermo, Waltham, MA), IL-10 (Thermo, Waltham, MA), and TNF-α (Thermo, Waltham, MA) in the culture supernatants were measured by ELISAs.

### Assessment of T lymphocyte differentiation and cytokine secretion *in vitro*

In total, 1 × 10^6^ lymphocytes derived from the spleens and lymph nodes of MHC-matched C57BL/6 mice were cocultured with a total of 1 × 10^5^ DCs or DCTP for three days. Subsequently, the T lymphocytes were collected and stained with rat anti-mouse monoclonal antibodies including anti-CD3e (Thermo, Waltham, MA), anti-CD4 (Thermo, Waltham, MA), anti-CD8a (Thermo, Waltham, MA), anti-IL4 (Cyto-Fast™ Fix/Perm Buffer Set, Biolegend, San Diego, CA), anti-IFN-γ (Cyto-Fast™ Fix/Perm Buffer Set, Biolegend, San Diego, CA), and anti-FOXP3 (True-Nuclear™ Transcription Factor Buffer Set, Biolegend, San Diego, CA), followed by FCM analysis. Moreover, cells stained with the anti-IL4 and anti-IFN-γ antibodies were pretreated with a Cell Activation Cocktail kit following the kit instructions (Biolegend, San Diego, CA). The culture supernatant was harvested, and the IFN-γ (Thermo, Waltham, MA), IL-2 (Thermo, Waltham, MA), IL-10 (Thermo, Waltham, MA), and TGF-β (Thermo, Waltham, MA) levels were measured by ELISAs.

### T cell purification

Splenocytes were isolated and stained with a PE-conjugated anti-CD3 antibody (Miltenyi Biotec, Bergisch Gladbach, Germany) by mixing and incubating for 15 minutes. Then, the cell pellets were washed and resuspended in 80 µL of buffer per 10^7^ total cells, mixed with 20 µL of anti-PE microbeads (Miltenyi Biotec, Bergisch Gladbach, Germany) and incubated for 15 minutes. Then, the T cells were isolated with a suitable MACS separator. The purified T cells were then cocultured with DCs or DCTP and analyzed by FCM three days later as described above.

### DC tracking

DCTP were labeled with the lipophilic tracer DID (Invitrogen, Inchinnan, UK) following the instruction manual. Briefly, DID (5 µL/mL) was coincubated with 1 × 10^6^ cells for 20 min at 37 °C. Then, the cells were washed three times and injected into mice with colitis via the tail vein. Twenty-four hours later, the spleen, mLN, colon, liver, kidney, heart, and testis were harvested and stored in liquid nitrogen. Then, the tissues were analyzed by generating frozen sections, DAPI staining (Beyotime, Shanghai, China) and fluorescence microscope observation.

### Statistical analysis

All the data are reported as the mean value ± standard error. Statistical differences between the treatment and control groups were evaluated by Sigma Stat (Systat Software, London, UK). Differences were considered statistically significant at *p*<.05 and highly significant at *p*<.01. Both parametric and nonparametric analyses were applied, in which the Mann–Whitney rank sum test (Mann–Whitney *U*-test) was used for samples with a non-normal distribution, and a two-tailed *t*-test was performed for samples with a normal distribution.

## Results

### DCTP exerted satisfactory treatment effects in mice with experimental colitis

**Figure 1.**
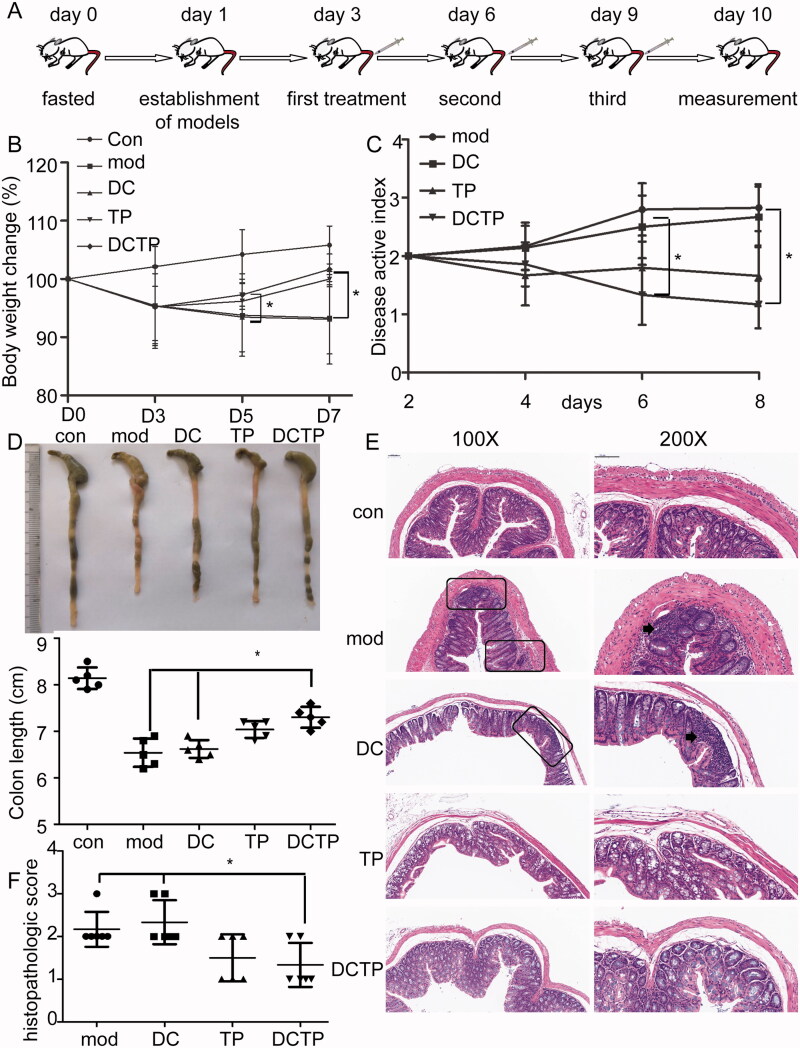
Examination of DCTP treatment in experimental colitis mice. C57BL/6 mice were treated with saline (Mod), DCs, DCTP, or TP three times intravenously at three-day intervals after a single enema of TNBS or saline (Con). (A) Schematic diagram of the dosing regimen for DCTP in C57BL/6 mice. (B) Body weight changes of colitis mice in each group on days 0, 3, 5, and 7. Mice treated with DCTP had a significant increase in weight on days 6 and 9 compared with those in the DC and Mod groups. (C) Disease active index (DAI) scores of mice in each group. Mice treated with DCTP had a lower DAI score than those in the DC and Mod groups. (D) Mice treated with DCTP had a significantly increased colon length. (E) H&E staining showed that DCTP reduced mucosal damage and inflammatory cell infiltration in the colon (the scale bar represents 100 µm). Arrowheads point to a microabscess and inflammatory cell infiltration. (F) The colon of mice in the DCTP group had a lower histological colitis score than that of those in the DC and Mod groups. Experiments were repeated three times (five mice per group, *n* = 15) (two-tailed *t*-test, *p<.05).

To investigate the therapeutic effects, DCs preconditioned with an optimal dose of TP (15 ng/mL, 24 hours) were injected intravenously (4 × 10^6^ DCTP per mouse) into MHC-matched C57BL/6 mice with experimental colitis three times at three-day intervals (Figure 1(A)). The weights of the mice in each group were recorded and analyzed. A decrease in body mass was observed after the establishment of colitis. The changes in body weight showed that mice in the DCTP group stopped losing weight after the first treatment on day 5. Significant differences (*p*<.05) were observed between the DC/DCTP and Mod/DCTP groups on days 6 and 9 (Figure 1(B)). The DAI score was calculated and showed significant decreases for the mice in the DCTP and TP groups compared with those in the Mod and DC groups (*p*<.05) (Figure 1(C)). A reduction in colon length was observed in the mice with UC compared with the normal mice, and the colon lengths of mice in the Mod, DC, TP, and DCTP groups were 6.54 ± 0.30 cm, 6.62 ± 0.19 cm, 7.04 ± 0.18 cm, and 7.30 ± 0.22 cm, respectively. Significant differences (*p*<.05) were observed among the DC/DCTP, Mod/DCTP, and Mod/TP groups (Figure 1(D)). Colon tissues from each group were stained with H&E and assigned histopathological scores by two double-blinded pathologists. Obvious ulceration, crypt abscesses, and inflammation were observed in the model mice. Compared with the Mod and DC groups, the DCTP and TP groups showed a reduction in inflammatory cell infiltration, a decreased number of crypt abscesses and reduced colonic mucosal damage (Figure 1(E)). The pathological scores of the DCTP and TP groups were significantly lower than those of the Mod and DC groups (*p*<.01, Figure 1(F)). These findings indicate that DCTP can result in an improvement in colonic inflammation and alleviation of local lesions in mice with experimental colitis.

### DCTP exhibited reduced toxicity in mice with experimental colitis

**Figure 2.**
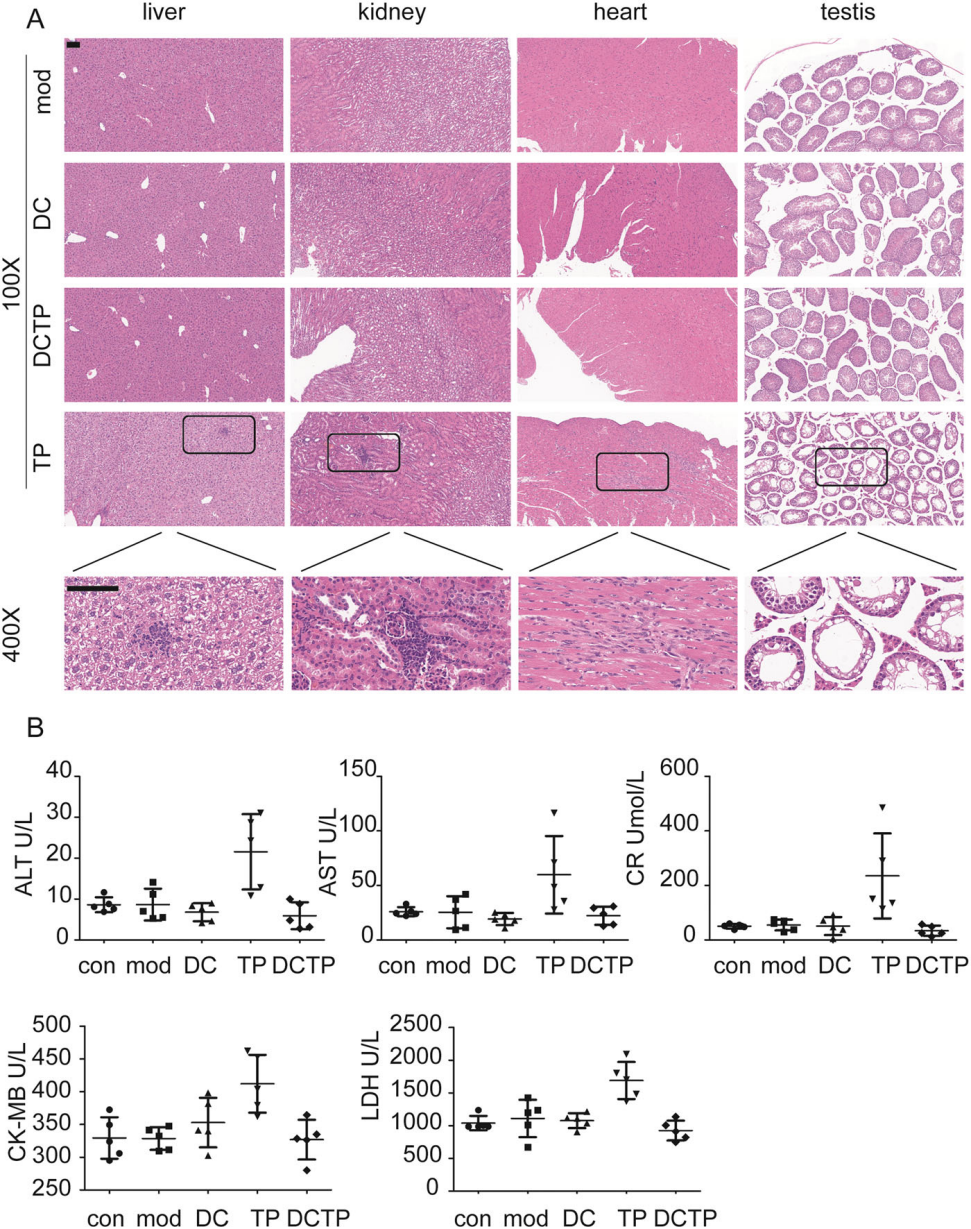
DCTP reduced the toxicity of TP in experimental colitis mice. (A) H&E staining of liver, kidney, heart, and testis tissues from the Mod, DC, DCTP, and TP groups. Obvious pathological damage was observed in the TP group. The liver tissues from the TP group showed hepatocellular hydropic degeneration, vacuolization, focal necrosis, and inflammatory cell infiltration. The kidney tissues from the TP group showed focal necrosis and inflammatory cell infiltration. The heart tissues from the TP group showed myocardial swelling, denaturation, cytolysis, and contraction band necrosis. The testis tissues from the TP group showed decreased spermatogenic cells and sperm, disarrangement of the spermatogenic tubule structure and intraepithelial vacuoles of varying sizes. There was no obvious injury in the DCTP group (the scale bar represents 100 µm). (B) Scatter plots of serum biochemical indicators including ALT, AST, Cr, CK-MB, and LDH showed liver, kidney, and heart injury in some of the mice in the TP group. Experiments were repeated three times in quintuplicate each time (*n* = 15).

TP may induce severe toxicity in multiple organs and tissues, which has been a main obstacle to its further application in the clinic. To examine whether DCTP reduced this toxicity, tissue samples from the most commonly affected organs, including the liver, kidneys, heart, and testis, were stained with H&E. Obvious pathological injuries were observed in some mice in the TP group. Liver injury including hepatocellular hydropic degeneration, vacuolization, focal necrosis, and inflammatory cell infiltration, was observed in three mice (*n* = 5). Kidney injury including focal necrosis and inflammatory cell infiltration was observed in three mice (*n* = 5). Heart injury, including myocardial swelling, denaturation, cytolysis, and contraction band necrosis, was observed in two mice (*n* = 5). Testis injury, including decreased spermatogenic cell and sperm numbers, disruption of the spermatogenic tubule structure and intraepithelial vacuoles of varying sizes was observed in two mice (*n* = 5) (Figure 2(A)). Moreover, the serum ALT, AST, Cr, LDH, and CKMB levels, which reflect liver, kidney, and heart injury, were measured. In the TP group, the levels of these indicators were remarkably increased in some mice (Figure 2(B)). However, no obvious damage was observed in the mice from the DCTP group, indicating that the generation and administration of DCTP is an effective approach to reduce the toxicity of TP.

### DCTP reshaped the immune microenvironment in mice with experimental colitis

**Figure 3.**
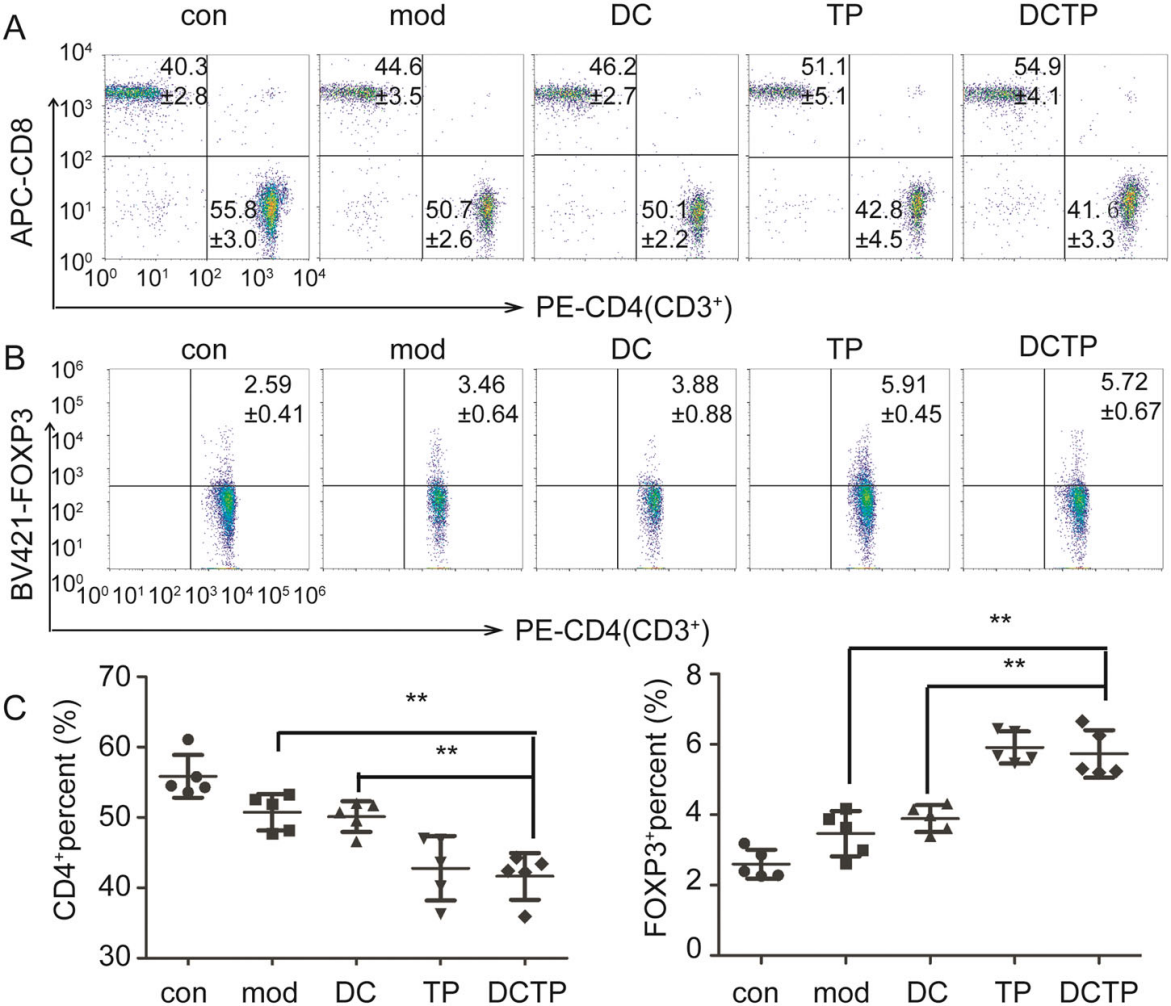
Analysis of T lymphocytes in the peripheral blood of mice. (A) Analysis of CD4^+^ T lymphocytes in peripheral blood from normal and experimental colitis mice treated with saline (Mod), DCs, DCTP, or TP (numbers represent mean ± SD). (B) Analysis of CD3^+^CD4^+^FOXP3^+^ Tregs in peripheral blood from normal and experimental colitis mice treated with saline (Mod), DCs, DCTP, or TP (numbers represent mean ± SD). (C) A significant decrease in the number of CD4^+^ T lymphocytes was detected in DCTP-treated mice compared with DC- and saline-treated mice. A significant increase in the number of Tregs was detected in DCTP-treated mice compared with mice in the DC and Mod groups. Experiments were repeated three times in quintuplicate each time (*n* = 15) (two-tailed *t*-test, **p<.01).

**Figure 4.**
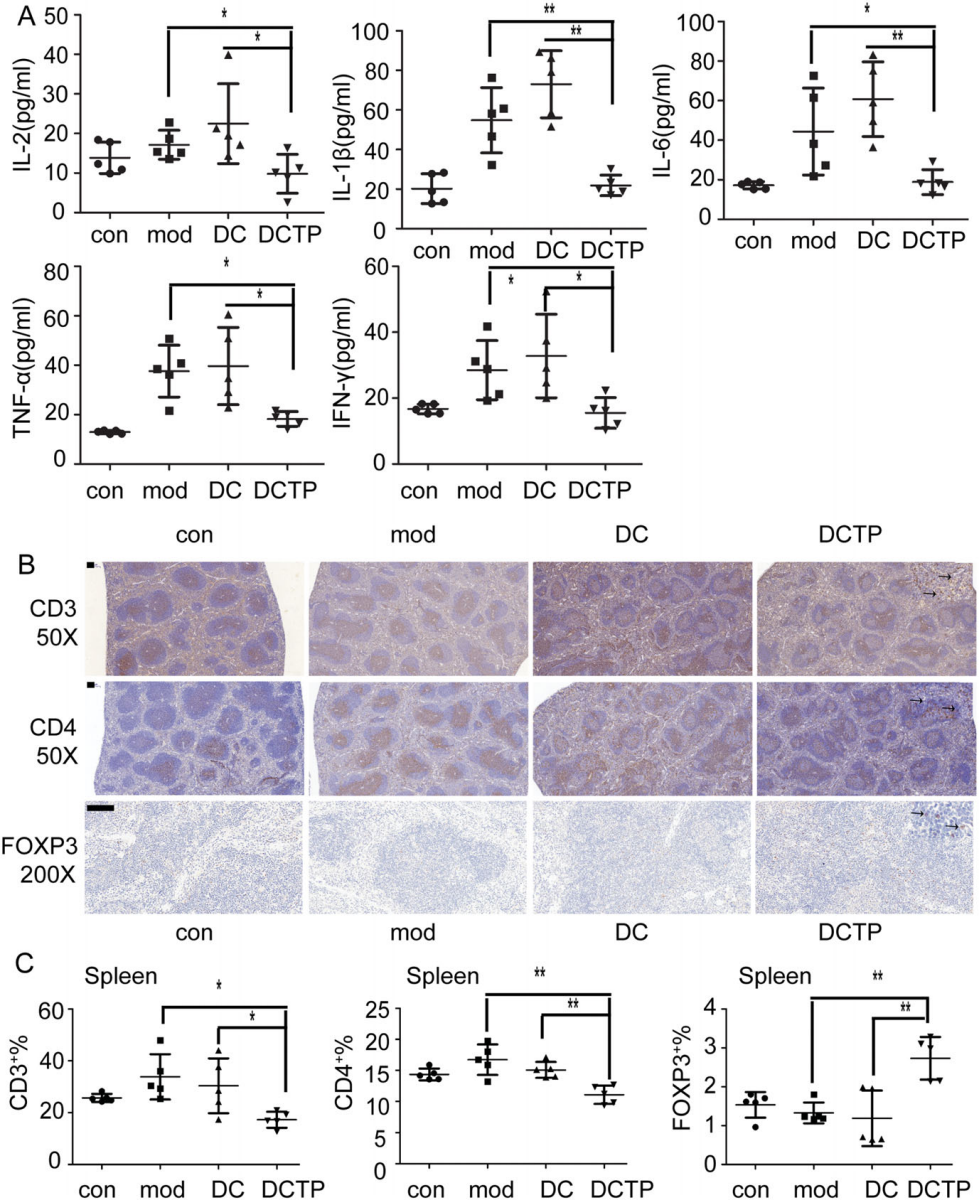
Analysis of serum cytokines and T lymphocytes in the spleen. (A) Measurement of IL-2, IL-1β, IL-6, TNF-α, and IFN-γ levels in the serum of normal (Con) and experimental colitis mice treated with saline (Mod), DCs, or DCTP. A significant difference was found between DCTP treatment and DC or saline treatment. (B) Immunohistochemical staining for CD3^+^ and CD4^+^ T cells and FoxP3^+^ Tregs in spleens from normal (Con) and experimental colitis mice treated with saline (Mod), DCs, or DCTP. T cells aggregated in splenic corpuscles in the Con and Mod groups and were primarily in the periarterial lymphatic sheath of the spleen in the DC and DCTP groups. A significant increase in FoxP3^+^ Tregs in the spleen was found in DCTP-treated mice compared with mice in the DC and mod groups (the scale bar represents 100 µm). (C) FCM analysis of the CD3^+^, CD4^+^, and FOXP3^+^ T cell percentages in the spleen showed an increase in Tregs and decreases in CD3^+^ and CD4^+^ T cells in the DCTP group. Experiments were repeated three times in quintuplicate each time (*n* = 15) (two-tailed *t*-test, *p<.05, **p<.01).

The systemic and local immune environments play a critical role in UC progression and DCTP therapy. Thus, T cell differentiation in the peripheral blood was first analyzed. The repeated administration of DCTP resulted in a significantly decreased number of CD4^+^T lymphocytes and an increased number of CD3^+^CD4^+^FOXP3^+^ Tregs compared with DC or Mod treatment (Figure 3(A–C)). Notably, analysis of serum cytokine levels in DCTP-treated mice showed dramatic reductions in the levels of IL-1β, IL-6, IL-2, IFN-γ, and TNF-α, a series of functional parameters of T cell immune and inflammatory responses (Figure 4(A)). These results demonstrated that DCTP decreased CD4^+^ T cell activation and induced Treg differentiation, indicating that the immune milieu was converted from an immunostimulatory environment to an immunoinhibitory environment, which is critical for the prognosis of UC patients.

The spleen, which is the most important peripheral immune organ, was evaluated by IHC to identify T cells. Importantly, T cells aggregated in the splenic corpuscle in the Con and Mod groups. However, in the DC and DCTP groups, T cells were primarily distributed in the periarterial lymphatic sheath, indicating that cell immunity was changed by the administration of the foreign DCs. Immunohistochemical staining of FoxP3 revealed higher numbers of Treg cells in the spleens of DCTP-treated mice (Figure 4(B)). Splenocytes were isolated and further analyzed by FCM, which consistently, showed decreased percentages of CD3^+^ and CD4^+^ T cells and increased percentages of FOXP3^+^ Tregs (Figure 4(C)).

**Figure 5.**
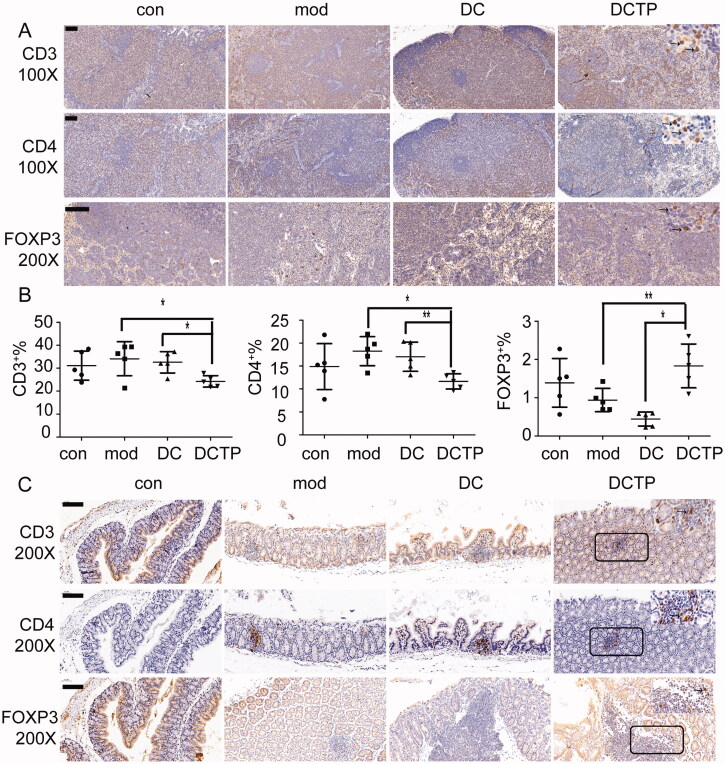
Analysis of lymphocytes in the mesenteric lymph nodes (mLN) and colon. (A) Immunohistochemical staining for CD3^+^ and CD4^+^ T cells and FoxP3^+^ Tregs in mLN from normal (Con) and experimental colitis mice treated with saline (Mod), DCs, or DCTP. Significant decrease in CD3^+^ and CD4^+^ T cells and increase in FoxP3^+^ Tregs were found in DCTP-treated mice compared with mice in the DC and mod groups. (B) FCM analysis of the CD3^+^, CD4^+^, and FOXP3^+^ T cell percentages in mLN showed an increase in Tregs and decrease in CD3^+^ and CD4^+^ T cells in the DCTP group. Experiments were repeated three times in quintuplicate each time (*n* = 15). (C) Immunohistochemical staining for CD3^+^ and CD4^+^ T cells and FoxP3^+^ Tregs in the colon of normal (Con) and experimental colitis mice treated with saline (Mod), DCs, or DCTP. Significant decreases in CD3^+^ and CD4^+^ T cells and an increase in FoxP3^+^ Treg infiltration were found in the colon of mice in the DCTP group compared with that of mice in the DC and Mod groups. Equivalent larger versions were shown in upper right corner for better observation (the scale bar represents 100 µm; two-tailed *t*-test, *p<.05, **p<.01).

To investigate whether the altered proportions of the T cell subpopulations improve the immune microenvironment in colonic lesions, we also examined the levels of T cell infiltration in the colon and mLNs. T cells in the mLN and colon were analyzed by FCM and IHC, respectively. Similar to the T cell distribution in the spleens, there were decreased percentages of CD3^+^ and CD4^+^ T cells and increased percentages of FOXP3^+^ Tregs in the mLN (Figure 5(A,B)). Immunohistochemical staining showed that T cells were prone to aggregate in micro abscesses or crypt abscesses in the colon. Compared with the DC and Mod groups, the DCTP group showed less CD3^+^ and CD4^+^ T cell infiltration in the colon tissues. Especially in normal colon tissue, the DCTP-treated mice had a significantly decreased number of CD4^+^ T cells. Moreover, FoxP3 staining revealed a trend of increased Treg infiltration in lesions in the DCTP group (Figure 5(C)), which was consistent with the results of the peripheral blood, spleen, and mLN. These findings supported the conclusion that DCTP can trigger a protective immune response and reshape the colonic immune microenvironment in mice with TNBS-induced colitis.

### TP converts DCs into tolerogenic DCs

**Figure 6.**
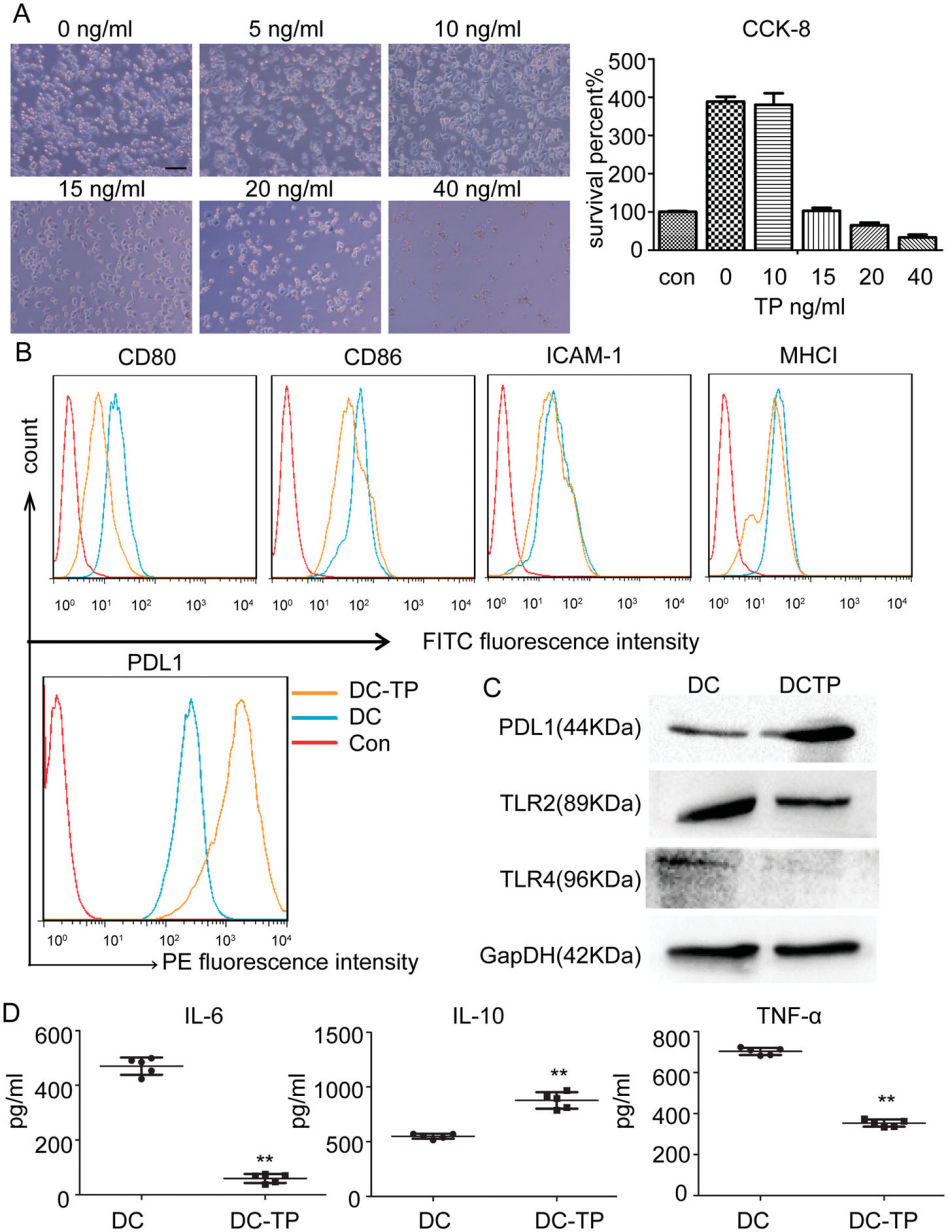
TP converts DCs into tolerogenic DCs. (A) Morphology of DCs treated with 0–40 ng/mL TP for 24 hours. Proliferation was inhibited, and apoptosis or death was consistently observed to be increased. A CCK-8 assay showed that TP at 15 ng/mL maintained a balance between DC proliferation and death. Con represents the initial cell number before cells were planked (con). (B) FCM analysis of the levels of the surface proteins CD80, CD86, ICAM-1, MHCI, and PDL1 in DCs or DCTP. DCs in Con group were unstained. Experiments were repeated three times in triplicate each time (*n* = 9). (C) Western blot examining the levels of PDL1 and Toll-like receptors 2 and 4 (TLR2 and TLR4) in DCs and DCTP. Total protein (20 µg) was loaded. (D) Measurement of the IL-6, IL-10, and TNF-α levels in supernatants from DCs or DCTP by ELISA. Experiments were repeated three times in quintuplicate each time (*n* = 15) (two-tailed *t*-test, **p<.01).

Given the DCTP-induced alternations to the protective immunoinhibitory environment *in vivo*, the immune mechanism was further examined. Why can DCTP induce an immunoinhibitory response? Microscopically, there were no obvious morphological changes in the DCTP as the concentration of TP increased from 0 to 15 ng/mL, but proliferation was inhibited. At TP concentrations higher than 20 ng/mL, obvious apoptosis or death was observed. A CCK-8 assay showed that 15 ng/mL TP maintained a balance between DC proliferation and death, so 15 ng/mL was chosen as the optimum concentration (Figure 6(A)). Surface molecules of DCs including ICAM-1, MHCI, and costimulatory factors, play essential roles in DC function. Notably, DCTP had lower expression levels of CD80, CD86, ICAM-1, and MHC I than untreated DCs (Figure 6(B)), suggesting that TP inhibited DC maturation and activation. PD-L1, also called B7-H1 or CD274, is another critical surface molecule on DCs that is related to immune inhibition or tolerance. We examined its expression with FCM and western blotting, and the results revealed significantly increased expression (Figure 6(B,C)). Toll-like receptors are receptors that recognize LPS and other antigens, and are related to UC pathogenesis and DC activation. Therefore, we examined the expression of two well-characterized TLRs, namely, TLR2 and TLR4. Notably, decreased TLR2/4 expression was observed in DCTP (Figure 6(C)). Further examination of cytokines secreted by DCs showed a significant increase in the level of the immunoinhibitory cytokine IL-10 and dramatic reductions in the levels of IL-6 and tumor necrosis factor α (TNFα), which are critical for DC function as antigen-presenting cells (Figure 6(D)). All the results above demonstrated that TP converts DCs into tolerogenic DCs.

### Tolerogenic DCs induce T cell-dependent immune tolerance by changing the differentiation of T lymphocytes

**Figure 7.**
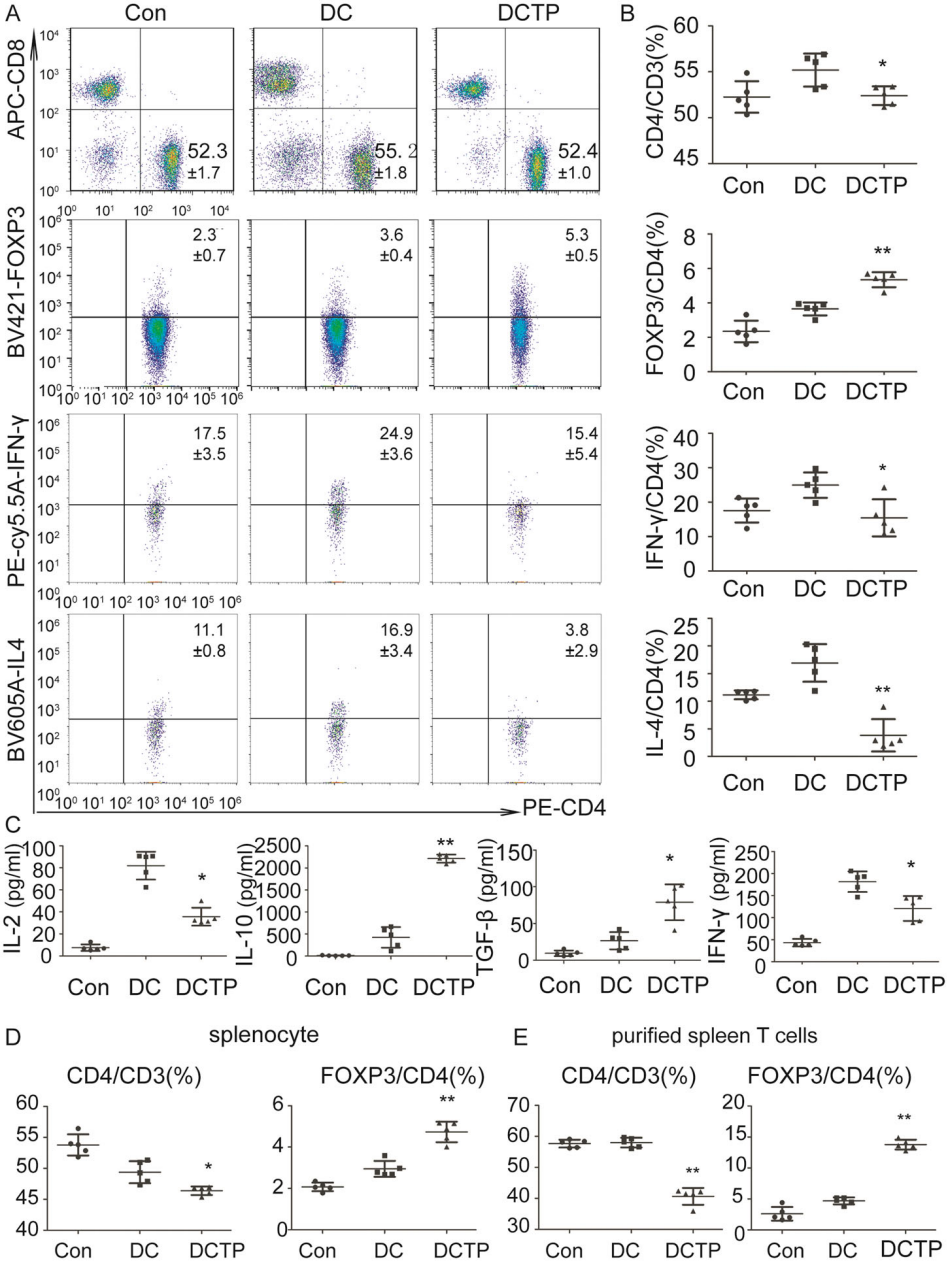
Tolerogenic DCs elicited T cell-dependent immunosuppression. Splenocytes or lymph node cells were isolated and cocultured with DCs or DCTP for 72 hours. Then, T cells and cytokines were analyzed. (A, B) FCM analysis of CD3^+^CD4^+^ T cells, CD3^+^CD4^+^FOXP3^+^ Tregs, CD3^+^CD4^+^IFN-γ^+^ Th1 cells, and CD3^+^CD4^+^IL4^+^ Th2 cells isolated from the lymph nodes. A significant decrease in the number of CD4^+^ T lymphocytes and an increase in Tregs were detected in DCTP cocultures compared with DC cocultures. IL4- and IFN-γ-positive T cells both showed significant reductions (numbers represent mean ± SD). (C) Measurement of IL-2, IL-10, IFN-γ, and TGF-β levels in supernatants from lymph node cells cocultured with DCs or DCTP by ELISA. (D) FCM analysis of CD3^+^CD4^+^ T cells and CD3^+^CD4^+^FOXP3^+^ Tregs isolated from the spleen. (E) T cells were purified from the spleen and cocultured with DCs or DCTP. FCM analysis of CD4^+^ T cells and Tregs showed more obvious changes consistent with those observed with mixed splenocytes. Experiments were repeated three times in quintuplicate each time (*n* = 15) (two-tailed *t*-test, *p<.05, **p<.01).

As antigen-presenting cells, tolerogenic DCs may directly affect the differentiation of T lymphocytes. To test this hypothesis, lymph node cells and splenocytes were isolated from C57BL/6 mice and cocultured with DCTP *in vitro* to mimic the immune response *in vivo*. Notably, DCTP significantly reduced the percentage of CD4^+^ T lymphocytes from lymph nodes compared with DCs. Regarding immunoinhibitory Tregs, the percentage of CD3^+^CD4^+^FOXP3^+^ T cells was significantly increased (Figure 7(A,B)). Cells were further stained to assess IL4 and IFN-γ levels, and the cells were analyzed by FCM. Decreased percentages of IL4- and IFN-γ-positive cells were observed in the DCTP group, which indicated that DCTP inhibited Th1 and Th2 differentiation simultaneously (Figure 7(A,B)). Further examination of the cytokine levels in the cell culture supernatants of DCTP-lymph node cell mixed cultures indicated a significant increase in the levels of the immunoinhibitory cytokines transforming growth factor-β (TGF-β) and IL-10 and dramatic reductions in the levels of IL-2 and IFN-γ, which are related to T cell activation and immunotolerance induction (Figure 7(C)). These results demonstrate that TP converts DCs into tolerogenic DCs and then markedly reverses T cell differentiation to induce immune inhibition. Regarding splenocytes, CD4 and Treg differentiation was consistent with that of lymph node cells (Figure 7(D)).

The splenocyte and lymph node cells evaluated were all cultured as mixed cell populations. To analyze whether T cells play a critical role in immune tolerance, T cells were purified from splenocytes with magnetic activated cell sorting and cocultured with DCs. The results showed that CD4^+^ T cell and Treg differentiation observed in the purified populations was consistent with but more obvious than that observed in mixed cells (Figure 7(D,E)), which indicated that the DCTP-induced immune tolerance was T cell dependent.

**Figure 8.**
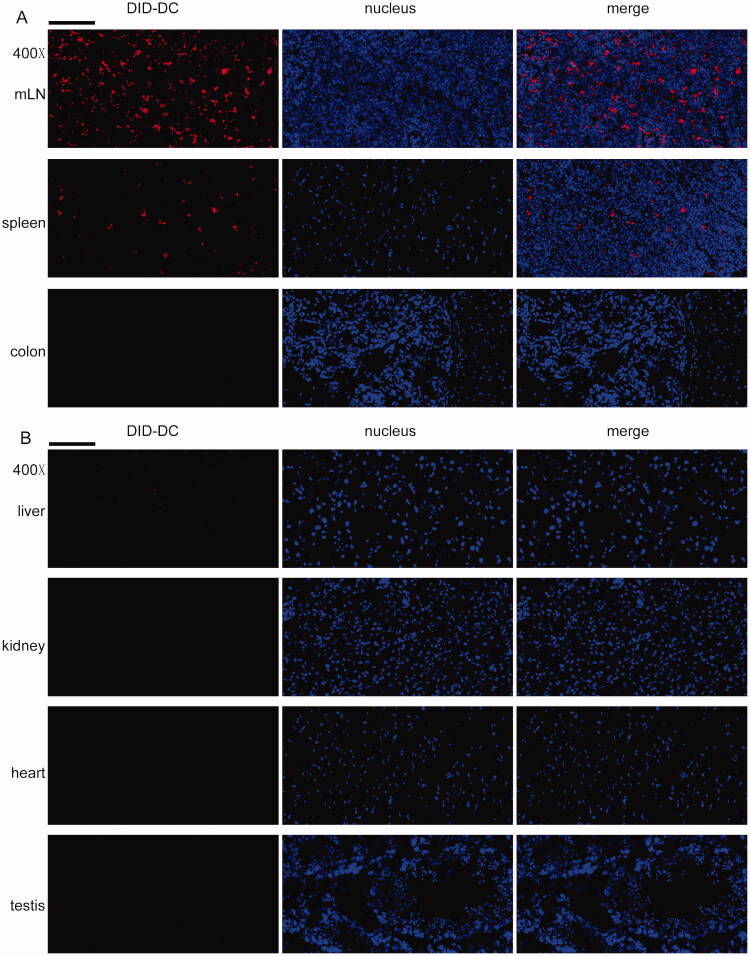
DCTP tracking in colitis mice. DCTP cells were labeled with DID and tracked by frozen sections, DAPI staining and fluorescence microscope observation. (A) DCTP was found in mLN and spleen but not colon. (B) No obvious DCTP was found in liver, kidney, heart, and testis (the scale bar represents 100 µm). Experiments were repeated three times.

Furthermore, DCTP were tracked in mice with colitis. DID-labeled DCTP were mostly found in the mLN and spleen but not the colon (Figure 8(A)), indicating that DCTP immunotherapy targeted immune organs or tissues to induce T cell-mediated immunosuppression, which verified our hypothesis described above. In the organs affected by TP toxicity, including the liver, kidney, heart, and testis, no DCTP (Figure 8(B)) were observed, which was associated with reduced DCTP toxicity.

## Discussion

UC is a chronic inflammatory disease with multifactorial pathogenesis, in which loss of immune tolerance plays a critical role (Tatiya-Aphiradee et al., 2018). Thus, immunoinhibitory drugs including corticosteroids and immune modulators, have been optimized to treat patients with moderate to severe UC. However, many patients do not respond to the limited number of therapeutic options available, and some suffer severe side effects (Toruner et al., 2008; Lichtenstein et al., 2012; Tatiya-Aphiradee et al., 2018).

In recent years, herbal therapy, an important component of traditional Chinese medicine, has attracted increasing attention in the treatment of inflammatory bowel disease, and a number of randomized controlled trials, including studies on aloe vera gel, *Triticum aestivum* (wheat grass juice) and *Andrographis paniculata* extract, have been completed (Ng et al., 2013). TP is the main active component of the *T. wilfordii* plant, which has been used for centuries in traditional Chinese medicine to treat autoimmune diseases such as rheumatoid arthritis. A number of studies have revealed the potent anti-inflammatory and immunoregulatory activities of TP (Matta et al., 2009; Han et al., 2012). A study by Zhang et al. (2016) demonstrated that after treatment with TP, the symptoms of UC in mice were significantly alleviated. Our previous study also showed that TP could regulate the composition of the gut microbiota, accelerate the recovery of the microbiota, and exert good therapeutic effects in mice with UC (Wu et al., 2019).

However, it has been demonstrated that TP exposure results in injury to various organs, including the liver, kidneys, testis, ovary, and heart in animals and humans (Li et al., 2014; Zhao et al., 2018), which seriously limits its clinical application. In recent decades, studies focused on reducing the side effects of TP have been conducted worldwide. Some approaches, such as structural modification or novel targeted delivery systems, have been examined to improve the tissue targeting of TP in order to reduce its toxicity (Xu & Liu, 2019).

Here, we sought to discuss the reason why we chose TP combined with DCs for UC therapy. DCs are a powerful tool that can be induced to differentiate into immunogenic DCs or tolerogenic DCs by many factors (Kushwah et al., 2010; Li et al., 2016). DC-based immunotherapy has been shown to be a promising strategy for treating a series of diseases (Chuma et al., 2015; Rao et al., 2016). Studies focused on the mechanism by which TP regulates immune responses have suggested that DCs might be the primary target of TP (Khan et al., 2009; Yuan et al., 2019). Thus, in this study, we combined DCs with TP to elicit an immunoinhibitory response and reduce TP toxicity. DCs were treated with TP *in vitro*, and then, DCTP were injected into mice to induce immune tolerance. Thus, direct exposure of other organs to TP was avoided.

Consistent with our expectations, our study demonstrated that DCTP could trigger an improvement in colonic inflammation and alleviate local lesion damage in mice with UC. Furthermore, no obvious damage was observed in the liver, kidneys, heart, or testis in the DCTP group, indicating that the DCTP approach reduced the toxicity of TP. The DCTP distribution showed no obvious DCTP infiltration in organs affected by toxicity, which contributed to its reduced toxicity.

Our further work showed that both *in vitro* and *in vivo*, DCTP altered T cell differentiation, decreasing the numbers of CD4^+^T cells and increasing the numbers of Tregs. Our findings also showed that DCTP reduced the Th1 and Th2 proportions simultaneously and that the immune tolerance induced was T cell dependent. Moreover, the immune milieu of the colon was also altered, with significantly reduced CD4^+^ T cell numbers and increased Treg infiltration in lesions. Tregs have been shown to play an essential role in preventing or limiting inflammation (Izcue et al., 2006; Yamada et al., 2016).

DC-T cell interactions are complex processes that include the activation of several signaling pathways. Signal 2, which includes a wide range of stimulatory or inhibitory interactions between receptors on the T cell surface and their ligands on the DC surface, determines the level of T cell activation (Arasanz et al., 2017). CD80/86-CD28 is one of the most important costimulatory interactions that induces T cells to acquire full proliferative and effector activities (Arasanz et al., 2017). Conversely, the PDL1-programmed cell death protein 1 (PD1) interaction strongly counteracts this signal transduction and plays a critical role in immune tolerance (Amarnath et al., 2011). ICAM-1 is one of the key adhesion molecules by which DCs adhere to the lymphatic endothelium and migration into afferent lymphatic vessels, and this process is important for immune activation (Jakubzick et al., 2006; Li et al., 2016). TLR signals, which induce anti-inflammatory gene activation and adaptive immune response in DCs, are critical for immune responses (Poltorak et al., 1998). Activation of signaling via the recognition of LPS by a TLR, specifically TLR4, increases intestinal inflammation and is closely associated with UC (Silva et al., 2016). Overall, the ability of DCs to activate or suppress immunity mainly depends on the expression of some key surface factors. Our study showed that TP changed the expression patterns of surface molecules and secreted cytokines by DCs, downregulating CD80/86, ICAM-1, MHCI, TLR2/4, TNF-α, and IL-6 expression and upregulating PDL1 and IL-10 expression; these effects converted DCs into tolerogenic DCs and were crucial for the maintenance of immune homeostasis and the control of autoimmune disorders (Li et al., 2016).

The purified T cell assay showed a consistent and more significant change in the immune response, demonstrating that the DCTP-induced immune inhibition was T cell dependent. Further study also found that DCTP were mainly distributed in the mLN and spleen but not the colon, indicating that DCTP-mediated immunotherapy acted on immune organs or tissues to elicit changes in T cell differentiation and distribution.

Our findings provide a strategy for not only treating UC but also reducing TP toxicity. DCTP also show promise for the treatment of other autoimmune diseases such as rheumatoid arthritis and a variety of skin diseases, including psoriasis, allergic contact dermatitis, polymorphous light eruption, erythema multiforme, atopic dermatitis, and pemphigus, that have been effectively treated with TP (Han et al., 2012; Yuan et al., 2019; Zhao et al., 2019a). Moreover, Zhang et al. (2013) found that infusion of DCTP into recipients before transplantation prolonged rat kidney allograft survival. Overall, TP combined with DCs may have excellent application prospects in the clinic.

Of course, there were some limitations to our study. First, our results were from a murine model. TNBS-induced experimental colitis models are widely used in drug tests and immunology research (Engel et al., 2010; Yang et al., 2016; Zhao et al., 2019b; Park et al., 2020). However, experimental colitis models have their own limitations in terms of comparisons with the systemic and colonic immune environments of patients in the clinic. Treatment compliance and the psychological state of patients also affect treatment. Moreover, the production and quality control of DCs is another difficulty for DC-mediated therapy. Further study is needed to elucidate the molecular and immune mechanisms. Thus, this study is the first step toward the development of DCTP therapy.
